# Adsorption of Orange II Onto Zn_2_Al–Layered Double Hydroxide Prepared From Zinc Ash

**DOI:** 10.3389/fchem.2020.573535

**Published:** 2020-11-30

**Authors:** Andra Tǎmaş, Ioana Cozma, Laura Cocheci, Lavinia Lupa, Gerlinde Rusu

**Affiliations:** ^1^Faculty of Industrial Chemistry and Environmental Engineering, University Politehnica Timisoara, Timisoara, Romania; ^2^“Coriolan Dragulescu” Institute of Chemistry, Timisoara, Romania

**Keywords:** layered double hydroxides, zinc ash waste, Orange II adsorption, equilibrium, kinetics, thermodynamics

## Abstract

The dye industry is one of the largest water consuming industries, and at the same time generates large quantities of wastewaters. The resulting wastewaters require proper treatment before discharge, because the dye contents have a negative effect on the water body and organisms present in it. The most efficient treatment method for water containing dyes is represented by adsorption processes. The challenge with these adsorption processes is to develop new, efficient, viable, and economic adsorbent materials. Therefore, in the present paper, the performance of Zn_2_Al-layered double hydroxide, prepared from an industrial waste (zinc ash) as a zinc source, was investigated in the Orange II dye adsorption process. The Zn_2_Al-layered double hydroxide prepared from secondary sources presents similar morphological and structural characteristics as those prepared from analytical grade reagents. The influence of initial dye concentration, adsorption time, solid:liquid ratio, pH, and temperature was evaluated in order to confirm the benefit of this waste valorization. A comparison with the reference Zn_2_Al-layered double hydroxide prepared from analytical grade reagents was performed and the results show that due to the small presence of impurities, the material prepared from zinc ash shows better adsorption capacities (q_max,exp_ = 42.5 mg/g at 293 K) than the material prepared from reagents (q_max,exp_ = 36.9 mg/g at 293 K), justifying the utilization of secondary sources for layered double hydroxides preparation. The proposed treatment process presents advantages from both economic and environmental protection point of view.

## Introduction

Synthetic dyes are used in many industries, such as the textile, plastics, leather, pharmaceutical, paint, and electroplating industries. Their intensive use leads to an increase of discharged effluents with dye content in water streams, representing an increased danger for environmental protection and human health (Jin et al., 2008; Hsiu-Mei et al., 2009; Kousha et al., 2012). In particular, the International Agency for Research on Cancer (IARC) classified Orange II dye as a category 3 carcinogen in humans (Pan et al., 2011). Their property of fast dispersion in water and their complex aromatic structure slow down the efficiency of the water treatment process, such as biological treatment and the oxidation processes (Abramian and El-Rassy, 2009; Hsiu-Mei et al., 2009; Jin et al., 2014; Asfaram et al., 2015; Ghaedi et al., 2015). From all the conventional processes used for treatment of water containing dye pollutants, adsorption proved to be the most efficient. If the formation of toxic sludge is avoided, it could be used for a wide range of water compositions—implying low operational costs (Bouhent et al., 2011; Mahmoodi et al., 2011; Kousha et al., 2012; Kaur et al., 2015). The most important factor which has the greatest influence on the efficiency of an adsorption process is represented by the adsorbent material type. An efficient adsorbent material must fulfill the following criteria: high adsorption kinetics, great selectivity, high adsorption capacity, thermal and chemical stability, and easy preparation. These properties are accomplished by layered double hydroxides (LDHs), also called hydrotalcite-like compounds, due to their distinctive characteristics; positively charged metal hydroxide layers and interlayer balancing anions which lead to large interlayer spaces, tunable chemical composition, and the reasonable number of exchangeable anions (Aguiar et al., 2013; Lafi et al., 2016; Mandal and Sandhya, 2019). In the last few years, in order to decrease the synthesis costs and also to reduce the quantity of waste discharged in the environment, attention has been focused on the LDH synthesis starting from secondary sources (Li et al., 2012; Murayama et al., 2012; Santini and Fey, 2012; Barik et al., 2014). Therefore, the novelty of this paper consists of the use of a Zn_2_Al-layered double hydroxide, prepared from an industrial waste zinc source (zinc ash), as the adsorbent material in the removal process of Orange II dye from aqueous solutions. The influence of initial dye concentration, time of adsorption, solid:liquid ratio, pH, and temperature was evaluated in order to confirm the benefit of this waste valorization. A comparison of the adsorption performance of the studied materials with a reference Zn_2_Al-layered double hydroxide, prepared from analytical grade reagents, was performed. The proposed method of using, as adsorbent materials, LDH obtained from an industrial waste, presents multiple advantages, such as: decreasing the waste discharged in the environment, minimizing the use of primary materials, and reducing costs of the adsorption process.

## Materials and Methods

**Figure 1:**
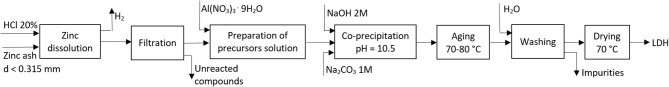
Schematic procedure of Zn_2_Al–W sample preparation from zinc ash.

All reagents utilized in this study were purchased from Merck (Germany) and were used as received. The layered double hydroxides (Zn_2_Al-CO_3_ - type) utilized in this study were prepared and characterized in a previous study (Cocheci et al., 2018). The sample Zn_2_Al – R was prepared by starting of analytical grade reagents and utilizing the co-precipitation at low oversaturation method, as described by Cavani et al. (1991). The sample Zn_2_Al – W was prepared starting from fine grained zinc ash (d < 0.315 mm), a waste produced by the hot-dip galvanizing process. The schematic procedure of the Zn_2_Al – W sample preparation is presented in Figure 1. After mixing the zinc ash with 20% HCl in order for zinc dissolution to occur, the zinc chloride solution was separated by filtration from unreacted compounds and utilized as a zinc precursor for the Zn_2_Al – type layered double hydroxide preparation, by utilizing the same co-precipitation method. The zinc chloride solution was mixed with a defined quantity of aluminum nitrate in order to obtain a molar ratio Zn^2+^ : Al^3+^ = 2 : 1 and a total molar concentration of the 1 M in 200 cm^3^ metal precursors solution. This solution was added drop by drop to a Na_2_CO_3_ 1M solution under vigorous magnetic stirring. The pH was kept constant to pH = 10.5 by adding a 2M NaOH solution and was monitored with an InoLab pH-meter. After total addition of metal precursors solution, the precipitate was aged at 70°C overnight, washed with distilled water until all impurities and unreacted compounds were removed, filtered, and dried at 70°C. The LDH samples were crushed and sieved in order to obtain particles with dimensions <90 μm.

**Figure 2:**
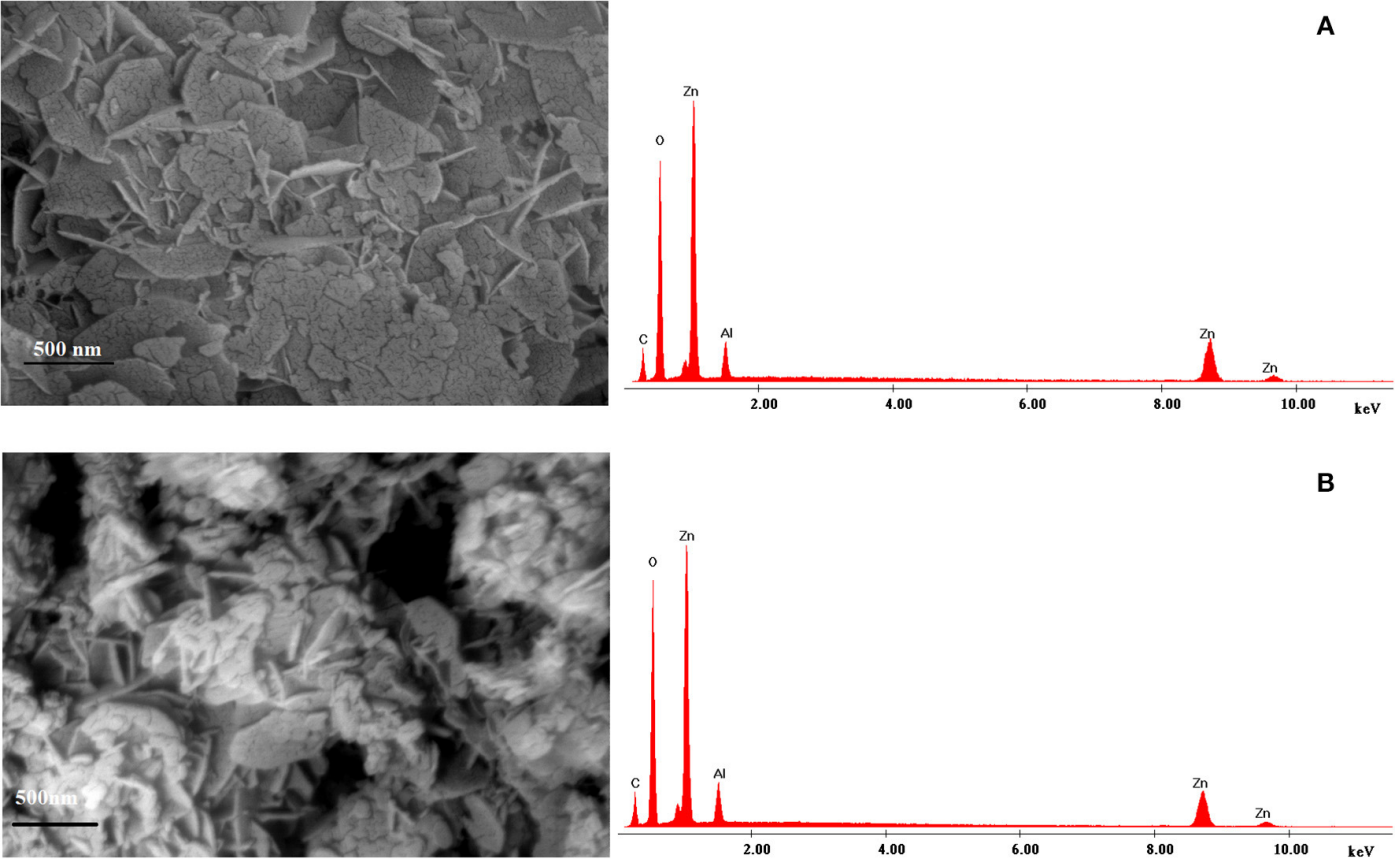
SEM images and EDX spectra for **(A)** LDH-W and **(B)** LDH-R.

The structural characterization of prepared LDH reveals that the sample prepared from zinc ash has similar characteristics to the sample prepared from analytical grade reagents Supplementary Table 1 presents the unit cell parameters for the two materials and the morphological characteristics (BET surface area and the pore volumes) for the synthetized samples (Cocheci et al., 2018). The SEM images are presented in Figure 2, together with the EDX spectra. From the SEM images of the materials, it is evident that the formation of highly agglomerated hexagonal plates with different sizes and good crystallinity, stacked with one another—this behavior being well-known for this type of materials synthesized by a co-precipitation method. In case of LDH-W (Figure 2A), due to the presence of a small quantity of impurities (Fe, Pb, Ca) from the zinc ash, an inhomogeneity of the surface and a smaller crystallinity is evidenced.

Orange II dye adsorption experiments were developed in batch mode in order to obtain information about the adsorptive behavior of the two materials. In order to study the benefit of waste valorization onto LDH, kinetic, thermodynamics and equilibrium studies were performed. The kinetic studies were performed studying the influence of different parameters such as: pH, solid:liquid ratio, initial dye concentration, and temperature at various stirring times upon the adsorption performance developed by the two studied LDHs. The adsorption characteristics were obtained by contacting a defined amount of adsorbent (Zn_2_Al – R or Zn_2_Al – W) with 50 cm^3^ solutions of different concentrations of dye (10 – 200 mg/L).

The adsorption capacity at equilibrium (q_e_) and at time t (q_t_) were calculated taking into account the mass balance of Orange II dye adsorbed onto the LDH surface:

(1) $$\begin{array}{l} q_{e}=\frac{\left(C_{0}-C_{e}\right)\cdot V}{m} \end{array}$$

where *q*_*e*_ is the adsorption capacity of adsorbent at equilibrium (mg/g), *V* the volume of the solution (L), *C*_0_ (mg/L) and *C*_*e*_ (mg/L) the initial and equilibrium concentrations of Orange II, and *m* the mass of adsorbent (g).

(2) $$\begin{array}{l} q_{t}=\;\frac{\left(C_{0}-C_{t}\right)\cdot V}{m} \end{array}$$

where *q*_*t*_ is the adsorption capacity of adsorbent at time t (mg/g), *V* the volume of the solution (L), *C*_0_ (mg/L) the initial concentration of Orange II, and *C*_*t*_ (mg/L) the concentration of Orange II at time t, and *m* the mass of adsorbent (g).

The structural characterization of exhausted materials after adsorption was performed by utilization of XRD and FTIR techniques. The X-ray diffractograms were recorded using a Rigaku Ultima IV X-ray diffractometer (40 kV, 40 mA) with Cu_Kα_ radiation. The Fourier-transform infrared spectra (FTIR) were recorded in the domain 400–4,000 cm^−1^ using Bruker Vertex 70 FTIR equipment. The concentrations of Orange II dye solutions were sprectrometrically determined at 480 nm using a Varian 100 UV-VIZ spectrometer.

## Results and Discussion

### Determination of pH_pzc_ for the Adsorbents

**Figure 3:**
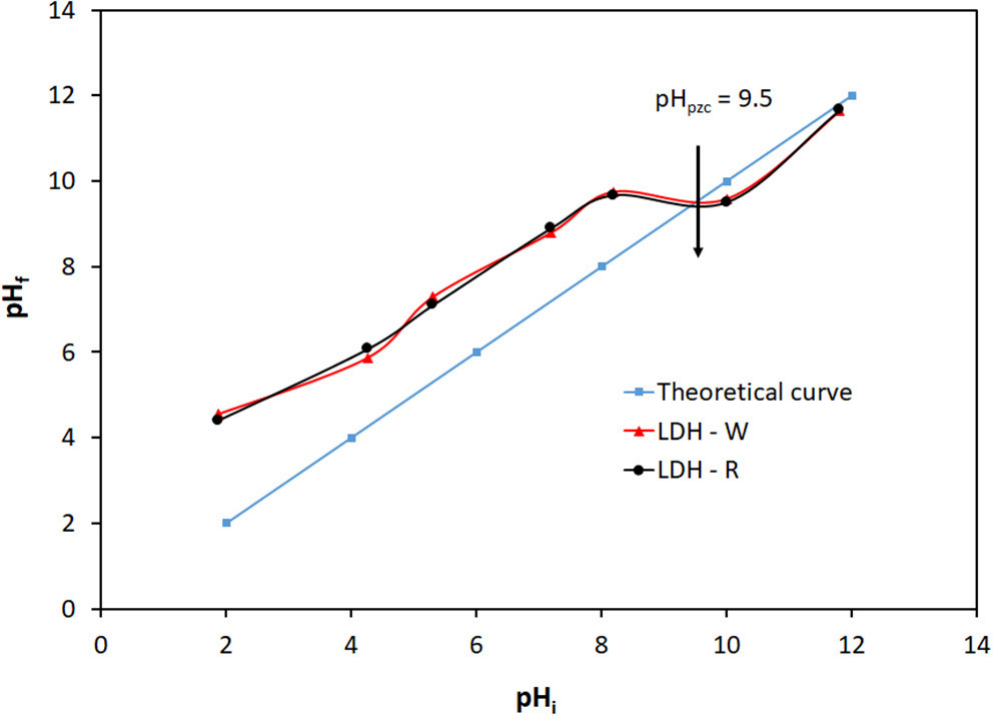
Determination of pH_pzc_ for the adsorbents.

Due to the fact that layered double hydroxides consist in positive layers of mixed metal hydroxides, the pH of working solutions plays an important role in the adsorption behavior of these materials. Depending on the value of the initial pH of the working solution, the surface of the materials can be either negatively charged, neutral at a particular pH, or positively charged. The value of pH at which the surface of the materials is neutral (the so-called pH of point of zero charged pH_pzc_) was determined by contacting 0.0050 g of solid materials with 50 cm^3^ of solutions of HNO_3_ or NaOH with defined pH_i_ values. After 24 h of mixing, the values of final pH (pH_f_) of the solutions were determined. By plotting pH_f_ = f(pH_i_), the pH_pzc_ can be determined as the intersection point of the obtained curve with the theoretical curve pH_i_ = pH_f_. The pH of point of zero charged for the studied materials is presented in Figure 3.

From Figure 3 it can be observed that the behavior of the materials (LDH – R, prepared from analytical grade reagents and LDH – W, prepared from waste) is similar and the pH = 9.5 represents the point of zero charge of the surfaces for both materials. At pH values lower than pH_pzc_ = 9.5 the surface of LDH became positive due to the protonation effect, while at pH values greater than pH_pzc_ = 9.5 the surface of LDH became negative due to the deprotonation effect (Xu et al., 2008). From a practical point of view, if the initial pH of the working solution has values lower than pH = 4, or greater than pH = 12, the mixed hydroxides can be dissolved.

On the other hand, the Orange II dye exists in aqueous solutions in various forms (Bourikas et al., 2005), but in the pH range of 2 – 9 only the partial ionized species (HL^−^) is present; the fully ionized species being predominant at pH > 12 (Zaheer et al., 2019).

### Kinetic Studies

#### At Different pH Values

**Figure 4:**
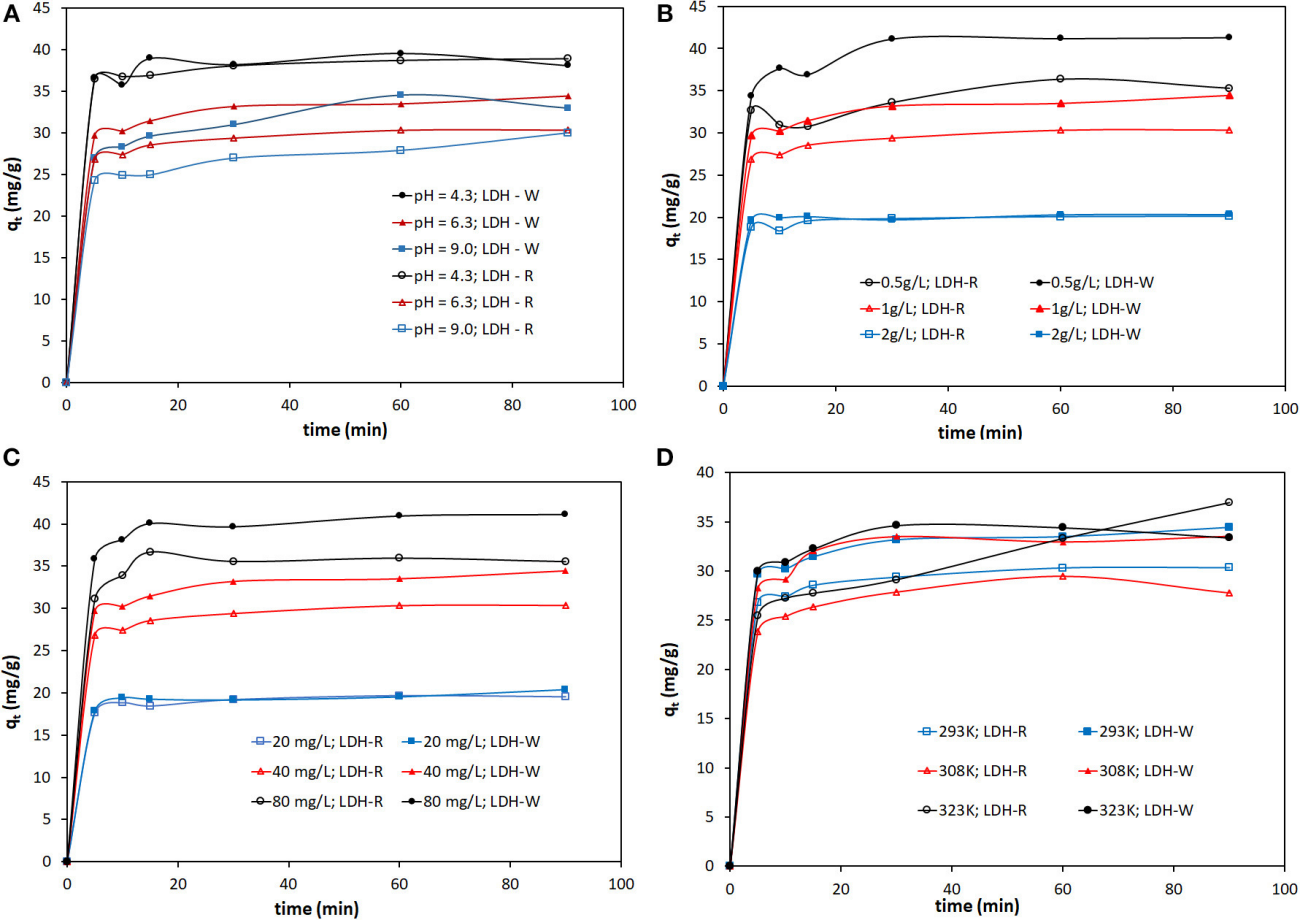
Influence of: **(A)** initial pH; **(B)** S:L ratios; **(C)** initial concentrations of dye and **(D)** temperature on the Orange II adsorption process at different stirring times.

The studies about pH influence on the adsorption process were performed by starting with 40 mg/L dye solutions, 1 g/L solid:liquid ratio, and an experimental temperature of 20°C. The influence of pH on the adsorption process is presented in Figure 4A.

It is evident that acidic conditions are favorable for the adsorption capacity of materials. As the pH of the dye solution increases, the adsorption capacity decreases. Comparing the sample prepared from analytical grade reagent (LDH – R) and that prepared from waste (LDH – W), at pH = 4 and after 90 min of adsorption, the adsorption capacities are similar. At initial pH = 6.3, the LDH prepared from waste (sample LDH – W) adsorbed better than the sample LDH – R (prepared from analytical grade reagents). We decided to run the following experiments at natural pH of initial Orange II solutions (5 < pH < 7) for practical convenience. It can also be observed that at each pH and for both studied adsorbents, the quantity of the adsorbed dye takes place very fast in the first 5 min of stirring. After that, the increase of the adsorption capacity is not so significant.

#### At Different Solid:Liquid Ratios

To study the influence of the quantity of adsorbent material on the removal of Orange II dye, different amounts of LDH were added, ranging between 25÷100 mg, into the flask containing 50 mL of dye solution. The experiments were performed at pH ~6.3, temperature 293K and the initial dye concentration of 40 mg/L. The resulting suspensions were stirred in a Julabo SW 22 multishaker at 200 rot/min. The samples were removed from stirring at predetermined time intervals and solids were removed by filtration and the filtrate was analyzed to establish the residual dye concentration. The results are shown in Figure 4B.

It is evident that the increase in the adsorbent dose lead to the decrease of the quantity of adsorbed dye. In case of the highest S:L ratio there is not a great difference between the adsorption capacity developed by the studied adsorbent. At a lower S:L ratio the adsorption capacity developed by LDH-W is higher than the adsorption capacity developed by LDH-R. Because the main purpose is to decrease the treatment costs, it is more efficient to use a lower S:L ratio. Therefore, further studies were performed using 1 g of LDH for the treatment of 1 L of aqueous solutions containing Orange II. By studying the second parameters, solid:liquid ratio, at different stirring times, the same conclusion can be drawn—that the main quantity of dye is adsorbed in the first 5 min of stirring. At higher stirring time, the adsorption capacity presents a slow increase, regardless of working conditions.

#### At Different Initial Concentrations of Dye

The effect of stirring time upon the adsorption performance was also studied using three different initial concentrations of dye (20, 40 and 80 mg/L). The experiments were performed at pH ~6.3, temperature 293K, and the dosage of adsorbent 1 g/L. The results are shown in Figure 4C.

The difference between the adsorption capacities developed by the two studied adsorbents is more relevant when it is used at higher initial concentrations of Orange II in solution. The presence, in the LDH structure (in case of the LDH-W, obtained from waste), of impurities (such as: Fe, Ca, Pb) lead to the decrease of its crystallinity, conferring inhomogeneity to the adsorbent surface and increasing the number of available sites for dye adsorption (Cocheci et al., 2018). At the same time, the LDH obtained from waste presents larger pores. All of this leads to the fact that LDH-W develops much higher adsorption capacity than LDH-R in the removal process of Orange II from aqueous solutions.

The performance of the studied materials developed in the adsorption process of Orange II from aqueous solutions was compared with the adsorption performance of other materials reported in specialty literature (Table 1). It can be observed that the Zn_2_AL-LDH prepared from secondary sources present better adsorption capacity than other similar materials studied for the removal of Orange II from aqueous solutions.

**Table 1:** The comparison between the adsorption capacities of similar adsorbents developed in the treatment processes of aqueous solutions containing Orange II.

**Adsorbent**	**q_***m*,**_ mg/g**	**References**
HDTMA coated zeolite	33.9	Jin et al., 2014
DMDOA-palygorskite (100% CEC equivalent modification)	38.61	Sarkar et al., 2011
2% Hexadecylammonium bromide (HDTMA)-modified zeolite	3.38	Jin et al., 2008
Spent brewery grains	28.54	Silva et al., 2004
Bottom ash	13.24	Gupta et al., 2006
LDH-R	35.75	Present paper
LDH-W	42.5	

#### At Different Temperatures

The kinetic studies were also performed at three different temperatures (293, 308, and 323 K) in order to determine the effect of temperature on the Orange II adsorption process, using LDH-R and LDH-W as adsorbent materials. The experiments were performed at pH ~6.3, the initial dye concentration of 40 mg/L and the dosage of adsorbent 1 g/L. The results are shown in Figure 4D.

The temperature increase lead to a slow increase of the quantity of dye adsorbed onto the studied materials. At the same temperature, the LDH obtained from waste developed a higher adsorption performance than the LDH obtained from the reagent.

It can be concluded that the equilibrium between the studied LDHs and the adsorbate is achieved quite quickly, regardless of working parameters. The largest quantity of Orange II is retained by the studied LDHs in the first 5 min of stirring, then the adsorption capacities of the material present a slow increase in time due to the decrease of the active site available for adsorption. After 15 min of contact between the two phases, the adsorption capacities of the studied materials are not influenced by the stirring time. It can be concluded that the studied layered double hydroxides are more efficient adsorbent materials for Orange II removal, than other adsorbents reported in literature due to the fact that in this case the equilibrium is achieved more quickly. In case of Orange II adsorption onto metal-Organic Framework (MOF) iron-benzenetricarboxylate (Fe(BTC)), the equilibrium is achieved between 80 and 140 min (García et al., 2014) and through adsorption onto phosphoric-acid modified clam shell powder after 90 min of stirring (Ma et al., 2013).

In order to investigate the mechanism of adsorption of Orange II onto LDH-R and LDH-W, kinetic models are used to test the experimental data. The experimental curves were fitted with the linear forms of the pseudo-first order (PFO) (Equation 3) (Georgin et al., 2016) and pseudo-second order (PSO) (Equation 4) (Hu et al., 2010; Plazinski et al., 2013; Georgin et al., 2016; Zaheer et al., 2019) models:

(3) $$\begin{array}{l} \ln\left(q_{e}-q_{t}\right)=lnq_{e}-k_{1}t \end{array}$$

(4) $$\begin{array}{l} \frac{t}{q_{t}}=\frac{1}{k_{2}\cdot q_{e}^{2}}+\frac{1}{q_{e}}\cdot t \end{array}$$

where k_1_ (min^−1^) and k_2_ (g mg^−1^ min^−1^) are the rate constants of the PFO and PSO models, respectively; q_e_ and q_t_ (mg g^−1^) are the equilibrium adsorption capacity and the adsorption capacity at time t (min), respectively.

**Figure 5:**
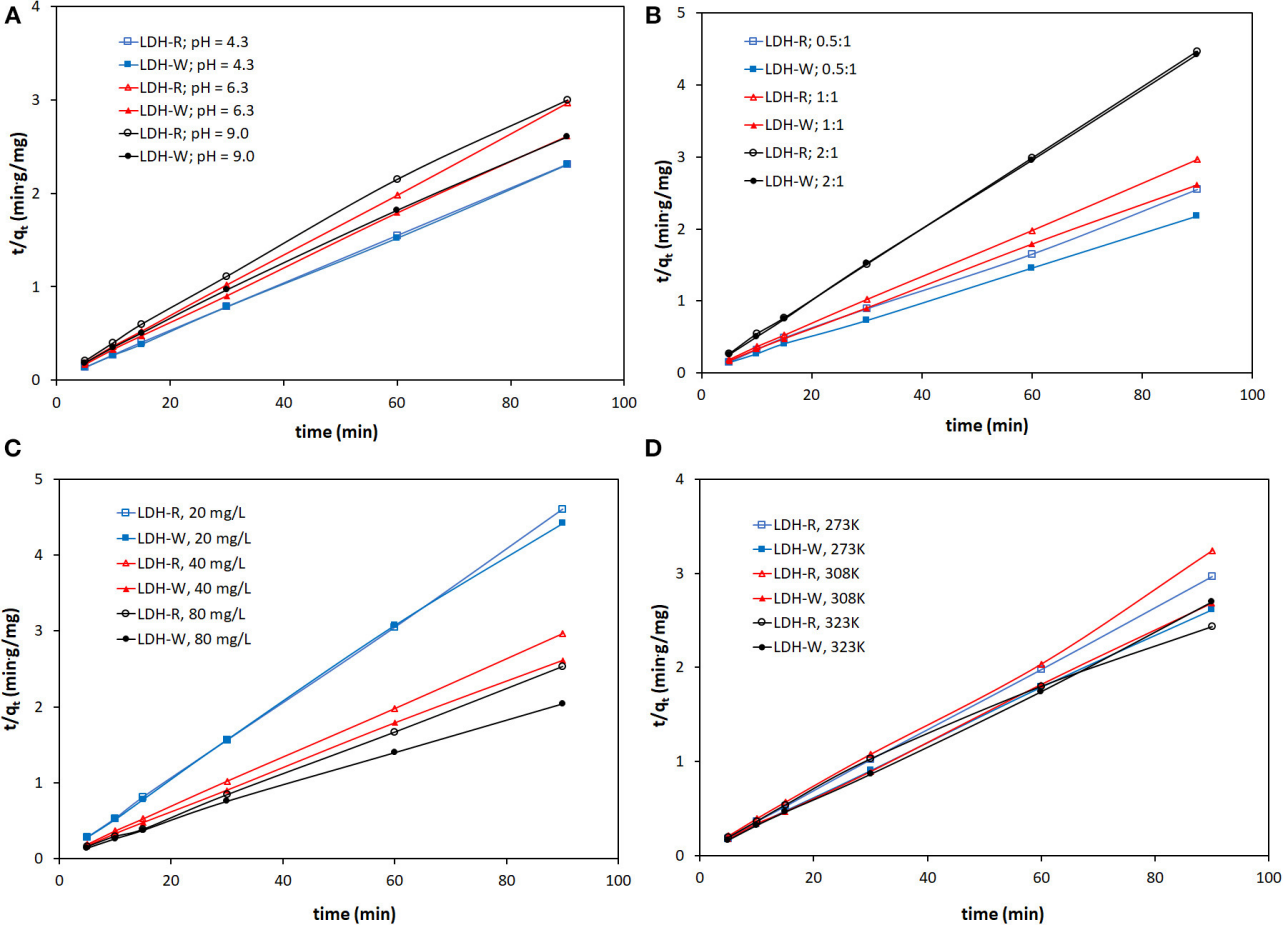
Pseudo-second-order kinetic models for adsorption of Orange II at: **(A)** different pH; **(B)** different S:L ratio; **(C)** different dye concentrations; **(D)** different temperatures.

The kinetic parameters k_1_ and q_e_ were determined from the slope and intercept of the linear plot ln(q_e_-q_t_) vs. t (figures omitted for the sake of brevity). For the PSO the graphical representations of t/q_t_=f(t) were used in order to determine the kinetic parameters k_2_ and q_e_ (Figure 5). The kinetic parameter together with the obtained correlation coefficients are presented in Supplementary Table 2.

The plots of t/q_t_ vs. t show good linearity, regardless of the working conditions, implying that the adsorption system follows the pseudo-second-order kinetic model. In all cases the correlations coefficients obtained for the fitting of the experimental data with the PSO model is close to unity compared with the correlation's coefficients obtained in case of the fitting of the experimental data with the PFO model. Furthermore, the q_e_ calculated from the linear plot of the PSO kinetic model are close to those experimentally determined. All of this suggests that the pseudo second order kinetic model describes the adsorption of Orange II onto the studied LDH, implying a chemisorption process.

### Equilibrium Studies

In order to determine the maximum adsorption capacities of the studied materials, equilibrium studies were performed with solutions containing initial concentration of Orange II between 10 and 100 mg/L, a S:L ratio of 1 g/L, at three different temperatures (293, 308, and 323 K). Due to the fact that, at 323 K, the equilibrium was not reached with the same starting dye solutions as in previous experiments, we performed the equilibrium studies with an initial concentration of Orange II between 40 and 200 mg/L.

The experimental data were fitted with the non-linear form of the Langmuir and Freundlich isotherms in order to describe the equilibrium between the adsorbent materials and Orange II. The non-linear mathematical expression of Langmuir model is represented in Equation (5) (Tran et al., 2016):

(5) $$\begin{array}{l} q_{e}=\frac{q_{max}\cdot K_{L}\cdot c_{e}}{1+K_{L}\cdot c_{e}} \end{array}$$

where q_max_ (mg/g) is the maximum saturated monolayer adsorption capacity under the given conditions and K_L_ (L/mg) is the Langmuir constant related to the affinity between an adsorbent and adsorbate.

The non-linear form of Freundlich isotherm is described by Equation (6):

(6) $$\begin{array}{l} q_{e}=K_{F}\cdot c_{e}^{\frac{1}{n}} \end{array}$$

where K_F_ (mg/g)(L/mg)^1/n^ is the Freundlich constant and 1/n (0 < n < 10) is a Freundlich intensity parameter.

**Figure 6:**
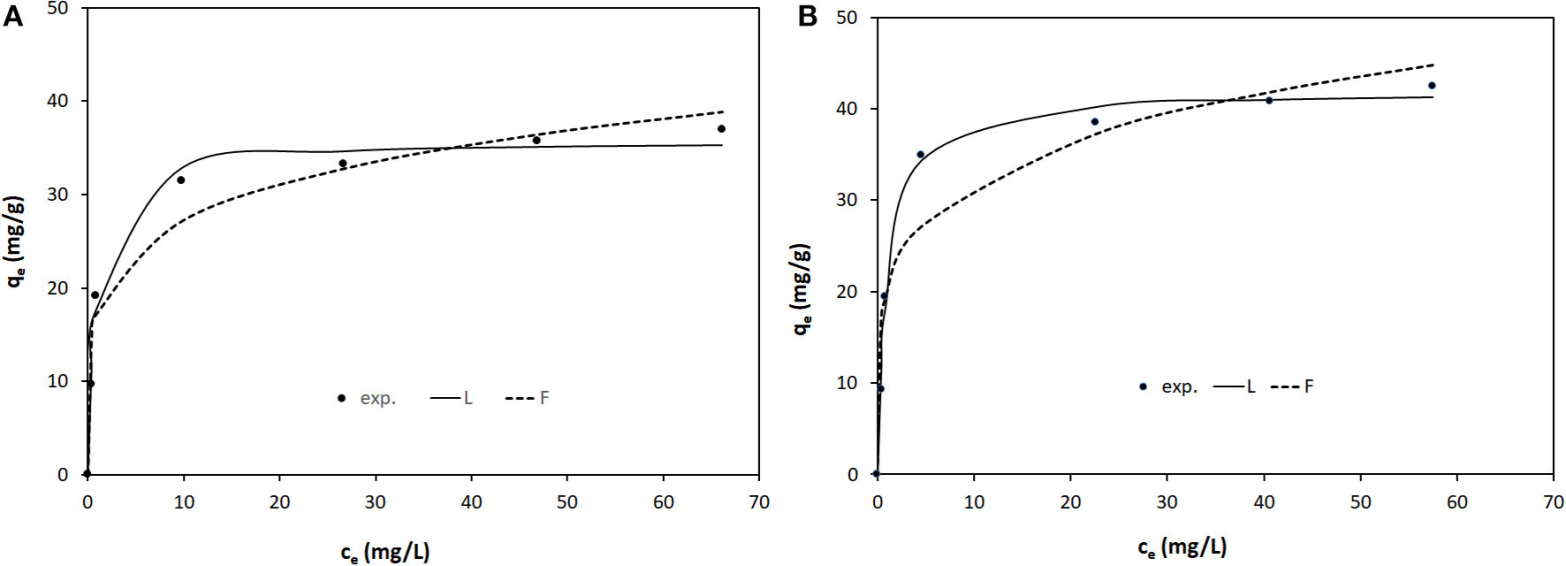
Equilibrium isotherms of Orange II adsorption onto: **(A)** LDH-R and **(B)** LDH-W at 293 K.

**Figure 7:**
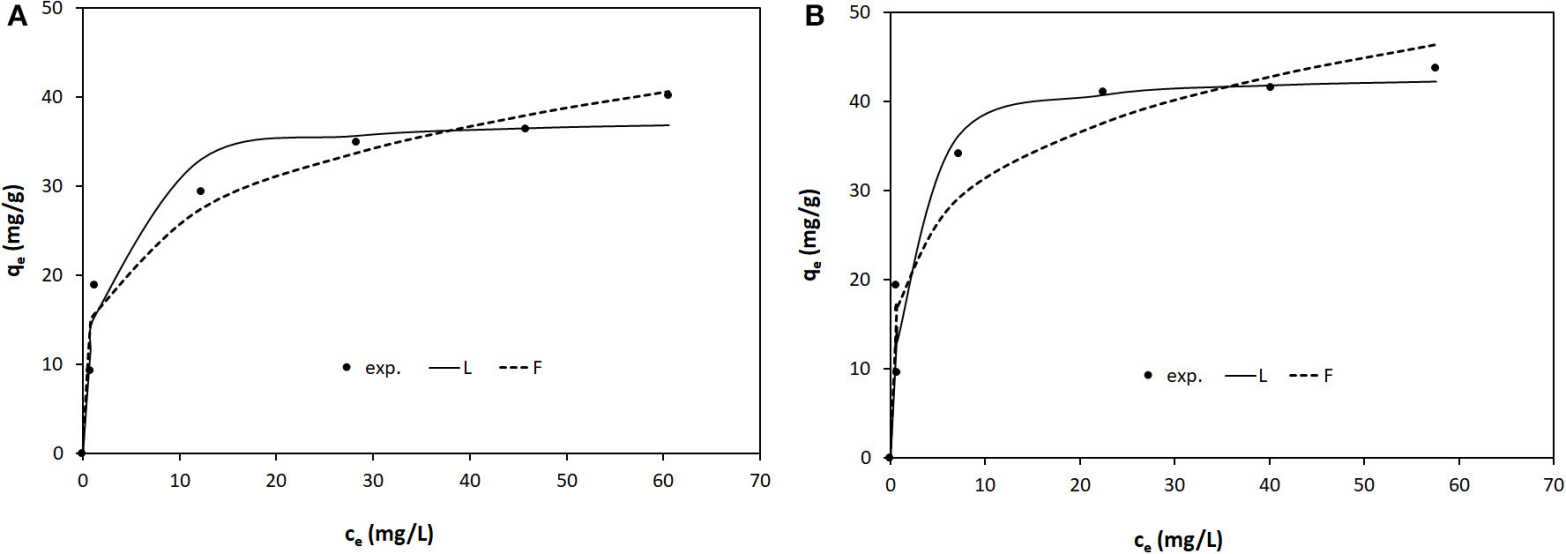
Equilibrium isotherms of Orange II adsorption onto: **(A)** LDH-R and **(B)** LDH-W at 308 K.

**Figure 8:**
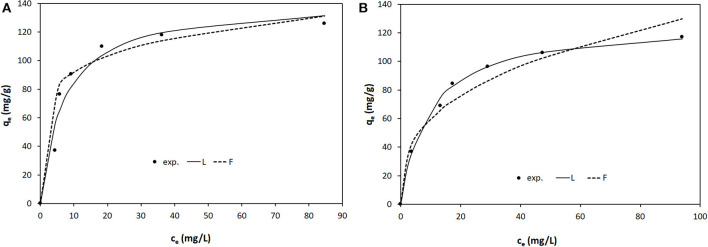
Equilibrium isotherms of Orange II adsorption onto: **(A)** LDH-R and **(B)** LDH-W at 323 K.

The equilibrium isotherms of Orange II adsorption onto LDH-R and LDH-W at the studied temperatures are shown in Figures 6–8. The equilibrium isotherms parameters, together with the obtained correlation coefficients, are presented in Table 2.

**Table 2:** Equilibrium parameters for Orange II adsorption onto LDH-R and LDH-W.

**Model**	**T(K)**	**LDH-R**				**LDH-W**			
		**q_max_, mg/g**		**K_*L*_, L/mg**	***R*^2^**	**q_max_, mg/g**		**K_*L*_, L/mg**	***R*^2^**
		**Exp**	**Calc**			**Exp**	**Calc**		
Langmuir	293	36.9	35.75	1.161	0.9814	42.5	42.00	0.991	0.9871
	308	40.17	37.93	0.551	0.9412	43.72	43.22	0.715	0.9201
	323	117	127.34	0.106	0.9949	126	142.02	0.147	0.9542
	**T(K)**	**LDH-R**				**LDH-W**			
		**K**_**F**_**, (mg/g)(L/mg)**^**1/*n***^		**1/n**	***R***^**2**^	**K**_**F**_**, (mg/g)(L/mg)**^**1/*n***^		**1/n**	***R***^**2**^
Freundlich	293	17.69		0.187	0.9111	20.11		0.198	0.8643
	308	14.88		0.245	0.9431	18.79		0.223	0.8831
	323	26.68		0.348	0.9388	34.34		0.324	0.8128
	**T(K)**	**LDH-R**				**LDH-W**			
		**q'_max_, mg/g**	**B**_**D**_ **x 10**^**7**^**, mol**^**2**^**/J**^**2**^	**E, kJ/mol**	***R***^**2**^	**q'_max_, mg/g**	**B**_**D**_ **x 10**^**7**^**, mol**^**2**^**/J**^**2**^	**E, kJ/mol**	***R***^**2**^
Dubinin-Radushkevich	293	33.87	1.305	1.957	0.9819	39.96	1.464	1.848	0.9863
	308	35.33	2.964	1.299	0.9681	38.83	1.696	1.717	0.8164
	323	77.01	3.11	1.268	0.8050	87.46	5.11	0.99	0.8335

The adsorption performance of the studied materials increases with the increase of the working temperatures. At 323 K the maximum adsorption capacities developed by LDH-R and LDH-W is 117 and 126 mg/g, respectively. These values are almost 3 times higher than the maximum adsorption capacities resulted at 293 K. Due to the fact that the isotherms present an S shape at 323 K the initial concentration of Orange II was increased until the equilibrium was reached. According to Giles and et al., this shape is obtained when the compounds containing a sulfonate group and an aromatic ring are absorbed vertically to the adsorbent surface, implying strong intermolecular forces (Giles et al., 1960). At a higher temperature, the diffusion and mobility of the Orange II particles are increased leading to a higher mass transfer rate from solutions to the surface of the studied LDHs. Furthermore, this behavior suggests that the adsorption of Orange II onto LDHs materials is a chemisorption. By increasing the temperature, the rate of the chemical reaction increases, the favorable intermolecular forces between the LDHs surface and dye are much stronger than those between adsorbate and solvent (Kaur et al., 2013; Gupta et al., 2017). The increase in adsorption capacities of adsorbents with an increase in temperature may also be attributed to the pore size enlargement (Demirbas et al., 2008; Banerjee and Chattopadhyaya, 2017).

It is observed that for both adsorbents the curves obtained from the two models reproduce, with sufficient accuracy, the experimental data. Also, for various temperatures and both models, the values of the correlation coefficient *R*^2^ are higher for LDH-R and, for the same type of adsorbent, these values are higher for the Langmuir model (Table 2). Furthermore, the maximum adsorption capacities developed by the studied adsorbent and experimentally determined are close to those calculated from the Langmuir isotherm, regardless of the working temperatures. These data indicate that the Langmuir model described the adsorption of Orange II onto the studied LDHs on the entire concentration range, suggesting that the adsorption takes place from a monolayer onto the adsorbent surface (Tran et al., 2016). The LDH obtained from waste developed a higher maximum adsorption capacity than the LDH synthesized from reagents (Table 2).

The strength of adsorption is indicated by the magnitude of the Freundlich constant (K_F_), which is the highest at the highest temperature (for both adsorbents) and for LDH-W (at the same temperature). For all the cases the Freundlich parameters 1/n present sub unitary values which indicates the affinity of Orange II for the studied adsorbents (Gupta et al., 2017).

In order to determine if the Orange II adsorption takes place due to a physisorption or a chemisorption process, the adsorption energy E was estimated by applying the Dubinin-Radushkevich (D-R) isotherm. The linearized form of this isotherm is given in Equation (7) (Hadi et al., 2010):

(7) $$\begin{array}{l} \ln\;q_{e}=\ln\;q_{m}-B_{D}\cdot \varepsilon^{2} \end{array}$$

where B_D_ is the D-R model constant (mol^2^/kJ^2^) and ε is the Polanyi potential (Equation 8):

(8) $$\begin{array}{l} \varepsilon =R\cdot T\cdot \ln\left(1+\frac{1}{c_{e}}\right) \end{array}$$

The adsorption energy E whose magnitude can provide information on adsorption mechanisms is determined from Equation (9), B_D_ being determined from the linear representation of lnq_e_=f(ε^2^):

(9) $$\begin{array}{l} E=\frac{1}{\sqrt{2\cdot B_{D}}} \end{array}$$

The D-R isotherm parameters are presented in Table 2. The low values of the adsorption energy confirm that the dye adsorption on both adsorbents is physically achieved. The maximum adsorption capacities calculated from the D-R isotherms present values lower than those experimentally obtained.

Due to the fact that the Langmuir isotherm best described the adsorption of Orange II onto the studied LDHs, the equilibrium experimental data were also fitted with its four linear forms which are presented in Equations (10)–(13) (Tran et al., 2016):

Langmuir linearization (type 1):

(10) $$\begin{array}{l} \frac{c_{e}}{q_{e}}=\frac{1}{q_{\max}}\cdot c_{e}+\frac{1}{q_{\max}\cdot K_{L}} \end{array}$$

Langmuir linearization (type 2):

(11) $$\begin{array}{l} \frac{1}{q_{e}}=\frac{1}{q_{\max}\cdot K_{L}}\cdot \frac{1}{c_{e}}+\frac{1}{q_{\max}} \end{array}$$

Langmuir linearization (type 3):

(12) $$\begin{array}{l} q_{e}=q_{\max}-\frac{1}{K_{L}}\cdot \frac{q_{e}}{c_{e}} \end{array}$$

Langmuir linearization (type 4):

(13) $$\begin{array}{l} \frac{q_{e}}{c_{e}}=K_{L}\cdot q_{\max}-K_{L}\cdot q_{e} \end{array}$$

The symbols have the same meaning as described before. The applicability of these isotherms was compared based on the error function values: correlation coefficient *R*^2^, chi-square test χ^2^ (Equation 14) (Hadi et al., 2010), average relative error ARE (Equation 15) (Li et al., 2017), and normalized standard deviation Δq (Equation 16) (Lv et al., 2007):

(14) $$\chi^{2}=\sum_{i=1}^{n}\frac{{\left(q_{e,\exp}-q_{e,calc}\right)}^{2}}{q_{e,calc}}$$

(15) $$\begin{array}{l} ARE=\frac{100}{n}\cdot {\sum_{i=1}^{n}\left|\frac{q_{e,calc}-q_{e,\;\exp}}{q_{e,calc}}\right|}_{i} \end{array}$$

(16) $$\begin{array}{l} \Delta q=100\cdot \sqrt{\frac{1}{n-1}\cdot {\sum_{i=1}^{n}\left(\frac{q_{e,\exp}-q_{e,calc}}{q_{e,\exp}}\right)}_{i}^{2}} \end{array}$$

The Langmuir parameters determined from its linear representations (figures omitted for the sake of brevity) are presented in Supplementary Tables 3, 4.

Each of the four linear forms of the Langmuir isotherm will result in different parameter estimates as shown in Supplementary Tables 3, 4. The adsorption of Orange II onto LDH-R and LDH-W adsorbents is well-represented by the Langmuir type I model with highest coefficient of determinations; *R*^2^ values as shown in Supplementary Tables 3, 4, indicate that there is strong evidence that the sorption of the chosen dye onto the prepared samples follows the Langmuir isotherm.

### Thermodynamic Studies

The thermodynamic parameters were calculated according to the following equations (Kaur et al., 2013; Banerjee and Chattopadhyaya, 2017; Gupta et al., 2017):

(17) $$\begin{array}{l} \Delta G^{o}=-RT\ln\;K_{d} \end{array}$$

(18) $$\begin{array}{l} \Delta G{}^{\circ}=\Delta H{}^{\circ}-T\Delta S{}^{\circ} \end{array}$$

(19) $$\begin{array}{l} \ln\;K_{d}=\frac{\Delta S{}^{\circ}}{R}-\frac{\Delta H{}^{\circ}}{RT} \end{array}$$

(20) $$\begin{array}{l} K_{d}=\frac{q_{e}}{C_{e}} \end{array}$$

where: ΔG° is Gibbs free energy (KJ/mol), ΔS° is the entropy and heat of adsorption (J/mol K), ΔH° is enthalpy (kJ/mol), T is the absolute temperature (K), R is universal gas constant (8.314 J/(mol·K)), and K_d_ is the distribution coefficient.

**Figure 9:**
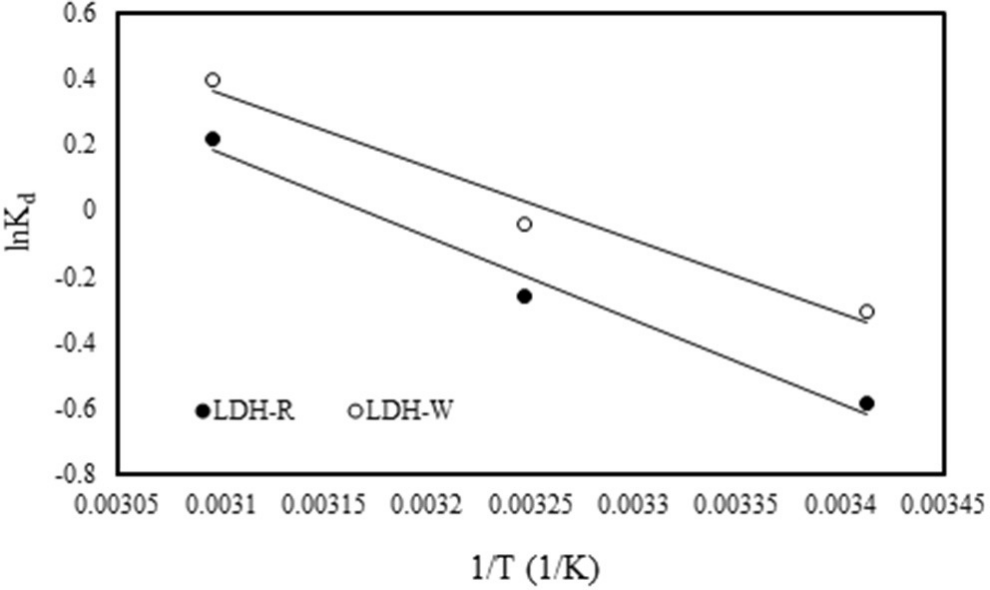
Effect of temperature on the adsorption of Orange II onto the studied materials.

ΔH° and ΔS° were obtained from the slope and intercept of van't Hoff plots of lnK_d_ vs. 1/T (Figure 9) and the resulted values, together with the Gibbs free energy calculated with Equation (17) and the correlation coefficients, are presented in Table 3.

**Table 3:** Thermodynamic parameters of Orange II adsorption onto the studied materials.

**Adsorbent**	**T (K)**	**R^**2**^**	**ΔG^**°**^ (kJ/mol)**	**ΔH^**°**^ (kJ/mol)**	**ΔS^**°**^ (J/mol K)**
LDH-R	293	0.9798	−0.126	19.3	66.3
	308		−1.12		
	323		−2.11		
LDH-W	293	0.9708	−0.163	17.3	59.6
	308		−1.06		
	323		−1.95		

The positive value of ΔH° thermodynamically substantiates the assumption that the adsorption of Orange II on the studied LDH is endothermic in nature. The feasibility and spontaneity of the adsorption process was confirmed by the obtained negative values of ΔG°. The positive value of ΔS**°** suggested an increase in randomness at the solid/liquid interface during adsorption of Orange II dye onto the studied materials. Adsorption is then likely to occur spontaneously at a high temperature because ΔH° > 0 and ΔS° > 0 (see Table 3) (Kaur et al., 2013; Banerjee and Chattopadhyaya, 2017; Gupta et al., 2017).

### The Structural Characterization of Exhausted Materials After Adsorption

**Figure 10:**
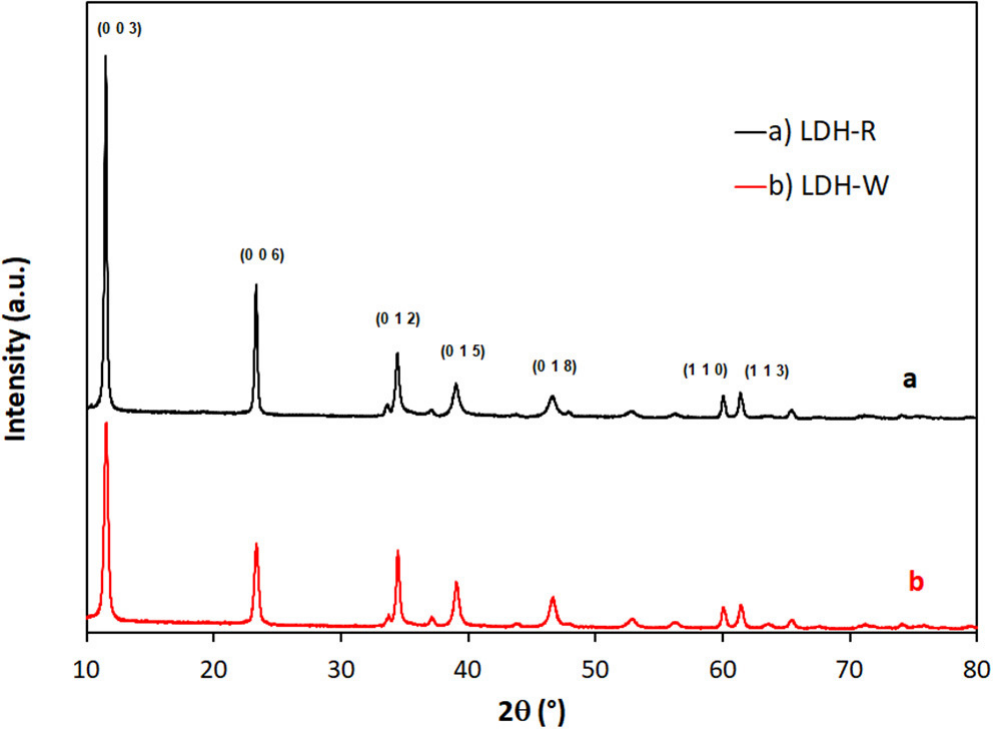
The XRD patterns of **(A)** LDH–R and **(B)** LDH–W after Orange II adsorption.

The XRD patterns of exhausted adsorbents are presented in Figure 10. The diffraction peaks of materials after Orange II dye adsorption are characteristic for layered double hydroxides with carbonate as an interlayer anion, since no modification in the position of the main peaks and basal spacing occurred. The basal spacing d_003_ and the d_110_ distances (cation-cation distance in the hydroxides layer) of the as-synthetized materials were d_003_ = 7.605 Å (d_110_ = 3.082 Å) for Zn_2_Al – LDH prepared from zinc ash and d_003_ = 7.598 Å (d_110_ = 3.080 Å) for Zn_2_Al – LDH prepared from analytical grade reagents (Cocheci et al., 2018). For the materials separated after Orange II adsorption, the basal spacing and d_110_ distances are: d_003_ = 7.593 Å (d_110_ = 3.079 Å) for LDH – W (prepared from waste) and d_003_ = 7.587 Å (d_110_ = 3.077 Å) for LDH – R (prepared from analytical grade reagents). Studies regarding Orange II intercalation in the interlayer space of layered double hydroxides find that the basal spacing became larger due to this intercalation (Geraud et al., 2007; Liu et al., 2007). The unchanged basal spacing in our case suggests that, due to the presence of a carbonate anion in the interlayer space of LDH (due to the strong electrostatic attraction and the hydrogen bonds that the carbonate creates with the hydroxides layer), the organic dye was adsorbed on the surface of the layered double hydroxides.

**Figure 11:**
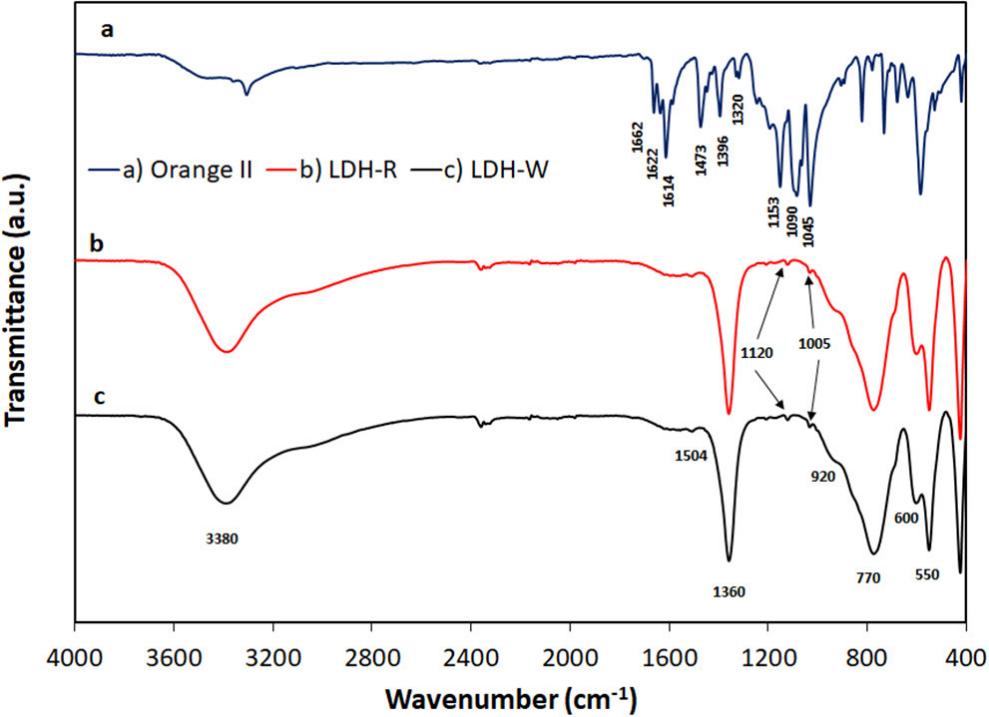
FTIR spectra of **(A)** Orange II dye; **(B)** LDH–R after adsorption; **(C)** LDH–W after adsorption.

The FTIR spectrum of Orange II (Figure 11) presents several absorption bands between 1,700 and 1,000 cm^−1^; the bands with maxima at 1,662 and 1,622 cm^−1^ correspond to a combination of phenyl ring vibrations with stretching of the C = N groups (Stylidi et al., 2004; Zhang et al., 2007). The band with a maximum at 1,614 cm^−1^ correspond to carbonyl (C = O) stretching vibrations and the maximum at 1,473 cm^−1^ are attributable to N = N stretching vibrations. The bands with maxima at 1,396 cm^−1^ and 1,320 cm^−1^ could be attributed to O – H bending vibrations (Stylidi et al., 2003). The absorption band with a maximum at 1,090 cm^−1^ could be attributed to the ν(N – N) stretching vibrations, and the maxima located at 1,153 cm^−1^ and 1,045 cm^−1^ correspond to the coupling between the phenyl ring and the ν_s_(SO_3_) mode of the sulfonate group (Zhang et al., 2007).

The FTIR spectra of exhausted adsorbents present the main absorption bands that are characteristic of layered double hydroxides but also show some small shoulders that correspond to the vibrations of sulfonate groups of Orange II dye (Figure 11). The main FTIR absorption bands of LDH – R (Figure 11B) and LDH – W (Figure 11C) could be attributed as follows: the strong band at 3,380 cm^−1^ is attributable to O – H stretching vibrations of Zn and Al hydroxides from the layer, the band with a maximum at 1,360 cm^−1^ could be assigned to the stretching ν_3_ mode of carbonate. The small shoulders at 1,504 cm^−1^ can be attributed to a lowered carbonate symmetry that activated the ν_1_ vibration mode of carbonate (Carja et al., 2001). The lowering of the carbonate symmetry activated the appearances of some weak shoulders corresponding to the ν_2_ and ν_4_ vibration mode of carbonate, visible at around 920 cm^−1^ and 600 cm^−1^, respectively (Kloprogge et al., 2002). The band located around 770 cm^−1^ and the band at around 550 cm^−1^ may be due to the Al – O or Zn – Al – O bond vibrations in the brucite-like layer (Cocheci et al., 2018). The small shoulders that are located at 1,120 cm^−1^ and 1,005 cm^−1^ could be assigned to the bands of sulfonate group of Orange II dye that are shifted to lower frequencies due to the interactions with the hydroxides layer (Geraud et al., 2007).

**Figure 12:**
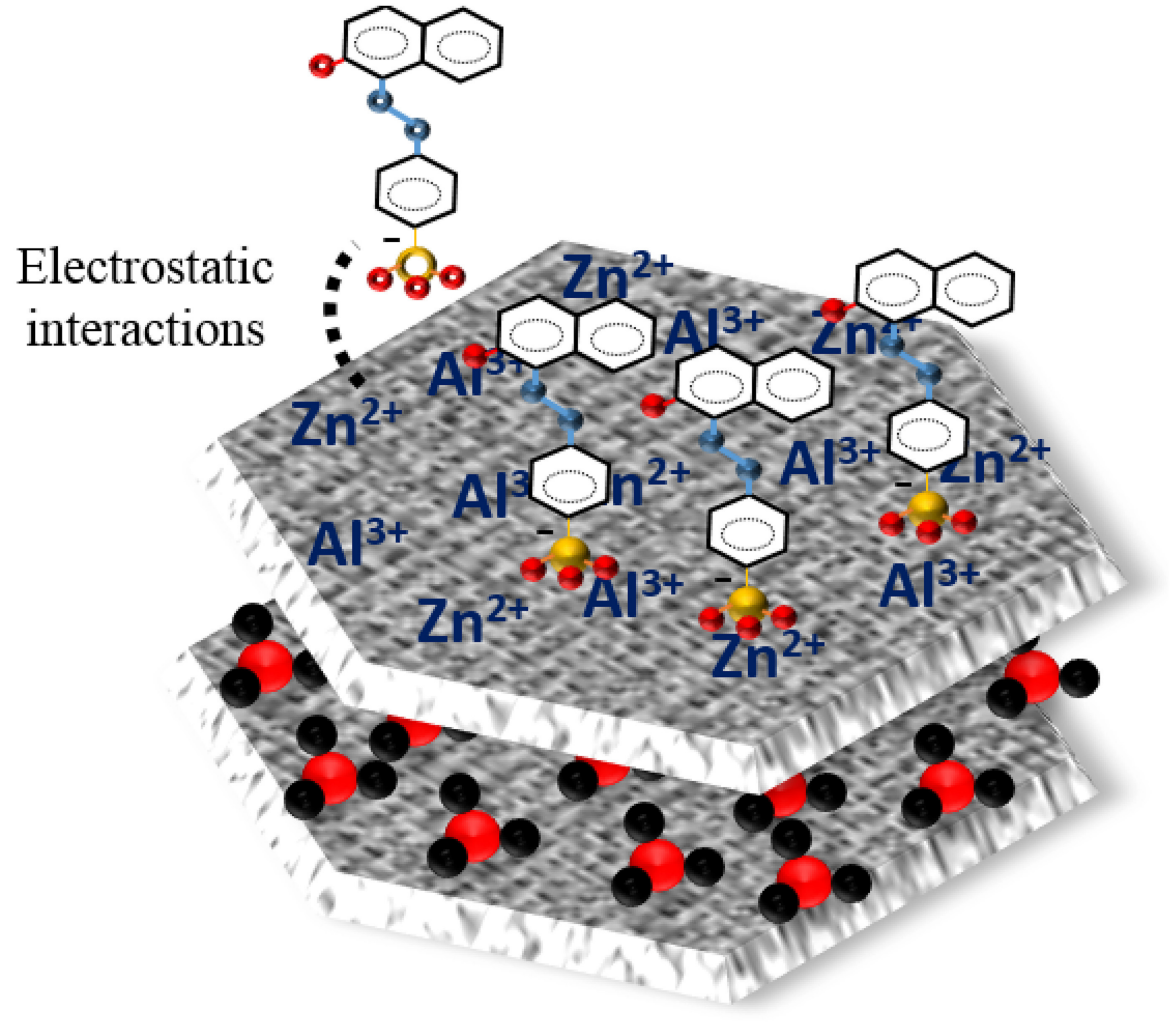
Schematic representation on Orange II adsorption onto surface of adsorbents.

The adsorption mechanism of Orange II onto the LDH-R or LDH-R adsorbents might involve two paths: external surface adsorption of dye *via* electrostatic interactions or replacement of carbonate anions from the interlayer region by the sulfonate group *via* anionic exchange (Lei et al., 2017; Srilakshmi and Thirunavukkarasu, 2019). From the structural characterization of exhausted materials after adsorption, it could be concluded that, in this case, the mechanism of Orange II adsorption onto the studied materials involves electrostatic interactions. This conclusion is in agreement with reports of other researchers who studied the adsorption of different anionic dyes on similar adsorbent materials (Geraud et al., 2007; Khosla et al., 2013; García et al., 2014; Jin et al., 2014). The schematic representation on Orange II adsorption onto the surface of adsorbents is presented in Figure 12.

## Conclusions

In the present paper, the performance of Zn_2_Al-layered double hydroxide, prepared from an industrial waste (zinc ash) as a source of zinc, and of Zn_2_Al-layered double hydroxide prepared from analytical grade reagents, as a reference, in the Orange II dye adsorption process, was investigated. The influence of initial dye concentration, time of adsorption, solid:liquid ratio, pH, and temperature was evaluated in order to confirm the benefit of this waste valorization. It was observed that the Orange II adsorption is strongly influenced by the studied parameters, the most efficient results being obtained at an initial pH value around 6, using a solid:liquid ratio of 1 g/L. The Orange II adsorption onto the studied LDH takes place very quickly, most of the dye content being adsorbed in the first 5 min, then the equilibrium is achieved in 15 min, regardless of the working conditions. The kinetic studies showed that the adsorption is described by the pseudo-second order and the equilibrium data presented a good fit with the Langmuir isotherm. The adsorption capacities increase with the increase of working temperature, and the thermodynamic parameters suggest the process of removal was spontaneous and endothermic. These results correlated with the results obtained from the structural characterization of exhausted materials after adsorption, through RX and FTIR, suggesting that the Orange II adsorption onto the studied materials occurs due to a chemisorption, implying strong intermolecular forces between the sulfonate group of the dye and the active sites of the adsorbent surface. For all the working conditions the LDH obtained from zinc ash shows better adsorption capacities than the material prepared from reagents, justifying the utilization of secondary sources for layered double hydroxides preparation. The proposed treatment process presents advantages from both an economic and environmental protection point of view.

## Data Availability Statement

The raw data supporting the conclusions of this article will be made available by the authors, without undue reservation.

## Author Contributions

LC and LL designed the study. AT and IC performed the experiments. LL and GR performed the instrumental analysis. AT, LC, and LL analyzed the data and wrote the paper with input from all authors.

## Conflict of Interest

The authors declare that the research was conducted in the absence of any commercial or financial relationships that could be construed as a potential conflict of interest.
