# Supplementary material for: Adsorption of Orange II Onto Zn2Al–Layered Double Hydroxide Prepared From Zinc Ash
Source: Front Chem. 2020 Nov 30;8:573535. doi: 10.3389/fchem.2020.573535 (PMC7793787; doi:10.3389/fchem.2020.573535)
Supplement: Supplementary file 1 [file Table_1.pdf]

Supplementary Material

Table S1. The main characteristics of the synthesized layered double hudroxides

Parameter / Sample	Zn/Al_{calc}	unit cell parameter *a* (Å)	unit cell parameter *c* (Å)	S_{BET} (m^2/g)	V_p (cm^3/g)
LDH-R	2.07	3.080	22.79	30.6	0.0682
LDH-W	1.98	3.082	22.82	21.8	0.0573

Zn/Al_{calc} – the calculated molar ratio R after chemical analysis; $a = 2d_{110}$; $c = 3d_{003}$

Table S2. Kinetic parameters for the adsorption of Orange II by LDH-R and LDH-W

**Kinetic model**		**LDH-R**			**LDH-W**		
	**pH**	**4.3**	**6.3**	**9**	**4.3**	**6.3**	**9**
PFO	$q_{e,calc}$ (mg g^{-1})	37.92	29.33	27.17	38.63	32.75	31.74
	k_1 (min^{-1})	0.646	0.469	0.411	0.578	0.443	0.341
	R^2	0.99682	0.99247	0.97288	0.9978	0.99893	0.99582
PSO	$q_{e,calc}$ (mg g^{-1})	39.21	30.77	30.3	39.37	34.72	35.09
	k_2 (g mg^{-1} min^{-1})	0.0372	0.0306	0.0121	0.0652	0.0205	0.0106
	h (mg g^{-1} min^{-1})	57.14	28.98	11.16	101.01	24.75	13.07
	R^2	1	0.9999	0.9977	0.9999	0.9998	0.9989
	$q_{e,exp}$ (mg g^{-1})	38.93	30.34	30.03	38.99	34.45	34.56
	**S:L ratio**	**0.5**	**1**	**2**	**0.5**	**1**	**2**
PFO	$q_{e,calc}$ (mg g^{-1})	33.34	29.33	19.64	39.96	32.75	20.07
	k_1 (min^{-1})	0.724	0.469	0.608	0.371	0.443	0.763
	R^2	0.97402	0.99247	0.99404	0.9884	0.98893	0.9992
PSO	$q_{e,calc}$ (mg g^{-1})	36.23	30.77	20.28	42.02	34.72	20.41
	k_2 (g mg^{-1} min^{-1})	0.0176	0.0306	0.0774	0.0192	0.0205	0.1312
	h (mg g^{-1} min^{-1})	23.15	28.98	31.85	33.90	24.75	54.64
	R^2	0.9988	0.9999	1	0.9998	0.9998	0.9999
	$q_{e,exp}$ (mg g^{-1})	35.31	30.34	20.13	41.29	34.45	20.33
	**C_0 (mg/L)**	**20**	**40**	**80**	**20**	**40**	**80**
PFO	$q_{e,calc}$ (mg g^{-1})	19.17	29.33	35.73	19.58	32.75	40.17
	k_1 (min^{-1})	0.499	0.469	0.399	0.486	0.443	0.431
	R^2	0.9969	0.9925	0.9976	0.9971	0.9889	0.9969
PSO	$q_{e,calc}$ (mg g^{-1})	19.72	30.77	35.71	20.37	34.72	44.84
	k_2 (g mg^{-1} min^{-1})	0.0747	0.0306	0.7129	0.0455	0.0205	0.0108
	h (mg g^{-1} min^{-1})	29.07	28.98	909.09	18.87	24.75	21.64
	R^2	0.9999	0.9999	0.9995	0.9991	0.9998	0.9991
	$q_{e,exp}$ (mg g^{-1})	19.72	30.77	35.71	20.37	34.72	44.84
	**T (K)**	**293**	**308**	**323**	**293**	**308**	**323**
PFO	$q_{e,calc}$ (mg g^{-1})	29.33	27.62	31.76	32.75	32.58	33.32
	k_1 (min^{-1})	0.469	0.369	0.269	0.443	0.365	0.431
	R^2	0.99247	0.9880	0.93313	0.98893	0.98921	0.99150
PSO	$q_{e,calc}$ (mg g^{-1})	30.77	28.57	37.88	34.72	33.90	33.90
	k_2 (g mg^{-1} min^{-1})	0.0306	0.0433	0.0051	0.0205	0.0279	0.0791
	h (mg g^{-1} min^{-1})	28.98	35.33	7.29	24.75	32.05	90.9
	R^2	0.9999	0.9982	0.9926	0.9998	0.9998	0.9992
	$q_{e,exp}$ (mg g^{-1})	30.34	27.77	36.97	34.45	33.53	33.37

Table S3. Langmuir parameters for Orange II adsorption onto LDH-R

T(K)	Model	q_{max}, mg/g	K_L, L/mg	R^2	χ^2	Δq, %	ARE, %
293		37.31	0.628	0.9987	4.096	18.92	15.68
308	Langmuir -type 1	40.65	0.305	0.9939	5.86	20.07	17.85
323		126.58	0.117	0.9982	0.86	5.27	2.75
293		37.17	0.971	0.9816	3.13	21.16	8.47
308	Langmuir -type 2	40.16	0.438	0.9154	2.48	14.36	11.69
323		107.53	0.173	0.9981	5.165	10.72	9.55
293		35.8	1.116	0.9445	0.484	7.1	5.72
308	Langmuir -type 3	38.53	0.398	0.9642	0.494	6.03	3.97
323		116.42	0.151	0.9591	2.22	8.57	5.83
293		36.27	1.054	0.9445	0.526	6.95	5.51
308	Langmuir -type 4	38.85	0.384	0.9642	0.479	6.15	4.48
323		118.5	0.145	0.9591	1.878	8.07	5.14

Table S4. Langmuir parameters for Orange II adsorption onto LDH-W

T(K)	Model	q_{max}, mg/g	K_L, L/mg	R^2	χ^2	Δq, %	ARE, %
293		42.92	0.71	0.999	1.646	11.14	7.95
308	Langmuir -type 1	44.25	0.61	0.9987	5.415	20.42	15.5
323		139.73	0.127	0.99	13.184	20.53	15.36
293		45.45	0.728	0.971	1.488	10.37	7.98
308	Langmuir -type 2	41.32	1.512	0.9799	0.615	7.32	4.33
323		222.22	0.058	0.9883	37.03	29.38	21.4
293		42.08	0.924	0.9201	0.645	9.44	6.28
308	Langmuir -type 3	45.58	0.373	0.996	0.058	1.98	1.43
323		132.82	0.236	0.9947	0.054	1.1	0.785
293		43.04	0.85	0.9201	0.744	8.84	6.39
308	Langmuir -type 4	45.63	0.372	0.996	0.059	1.98	1.38
323		132.98	0.235	0.9947	0.054	1.1	0.78

References

Cocheci, L., Lupa, L., Gheju, M., Golban, A., Lazău, R. and Pode, R. (2018) Zn–Al–CO_3 layered double hydroxides prepared from a waste of hot-dip galvanizing process. Clean Technol. Environ. Policy 20, 1105–1112. doi: 10.1007/s10098-018-1533-3
